# Neochetracin: An Unusual Chetracin-Type Epithiodiketopiperazine Derivative Produced by the Fungus *Amesia atrobrunnea*

**DOI:** 10.1021/acsomega.4c02424

**Published:** 2024-05-17

**Authors:** Esteban Charria-Girón, Christina Sauer, Dania García, Sherif S. Ebada, Yasmina Marin-Felix

**Affiliations:** † Department of Microbial Drugs, Helmholtz Centre for Infection Research, Inhoffenstraße 7, 38124 Braunschweig, Germany; ‡ Institute of Microbiology, Technische Universität Braunschweig, Spielmannstraße 7, 38106 Braunschweig, Germany; ⊥ Unitat de Micologia i Microbiologia Ambiental, Facultat de Medicina i Ciències de la Salut and IISPV, Universitat Rovira i Virgili, 43201 Reus, Spain; § Department of Pharmacognosy, Faculty of Pharmacy, Ain Shams University, 11566 Cairo, Egypt

## Abstract

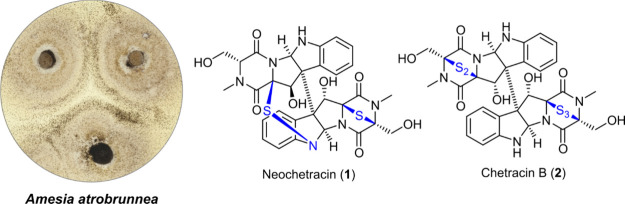

Epithiodiketopiperazines are a widely distributed class of secondary metabolites originating from an NRPS biosynthetic pathway and featuring diverse biological activities. In this study, the soil-borne fungus *Amesia atrobrunnea* FMR 19325 was found to produce a novel chetracin-like epithiodiketopiperazine, neochetracin (**1**), featuring a unique C-11a′-S-N cross-linkage, along with the known congener, chetracin B (**2**). Chemical structures were elucidated based on HR-ESI-MS and comprehensive 1D/2D NMR spectroscopic analyses. The relative configuration of **1** was distinguished based on a ROESY experiment while its absolute configuration remains undetermined. Chetracin B was found to be a more potent cytotoxic agent compared with its new congener. Compounds **1** and **2** also exerted strong antibacterial effects against the tested bacteria; however, our results suggested that the presence of the C-11a′-S-N cross-linkage in **1** resulted in the total or partial loss of activity against Gram-negative bacteria.

## 1 Introduction

The chemical diversity observed in fungal natural products emerges as a part of the ongoing evolutionary processes through which fungi adapt to their ecological niches.^1^ A fair example of this occurs with epidithiodiketopiperazines (ETPs), a family of fungal metabolites exerting potent biological activities arising from a varied range of molecular architectures.^2^ Generally, these compounds are synthesized by two modules of nonribosomal peptide synthetases (NRPSs) together with tailoring enzymes acting over the obtained core.^3^ Their chemical complexity has, for decades, challenged and inspired various synthetic approaches trying to mimic the creativity and the efficiency of fungi in building upon this class of compounds.^4^

Members of the order Sordariales, particularly those belonging to family Chaetomiaceae, are renowned for their numerous contributions across various fields, including agriculture, biotechnological applications, and food production.^5^ Chaetomiaceous fungi are currently the largest source of unique and structurally diverse metabolites in the Sordariales, with several of them possessing significant bioactivities.^5,6^ During our ongoing research to explore novel bioactive secondary metabolites from fungal species belonging to the order Sordariales,^6,7^ a rare chetracin-like ETP (**1**) and the known chetracin B (**2**) were isolated from the liquid culture of a chaetomiaceous soil-borne fungus, *Amesia atrobrunnea* FMR 19325. Neochetracin (**1**) represents a novel exemplar of this class of compounds possessing a unique unprecedented C-11a′-S-N cross-linkage. Herein, the isolation, structure elucidation, and biological activities of compounds **1** and **2** are described.

## 2 Results and Discussion

The secondary metabolites production of the fungus *Amesia atrobrunnea* FMR 19325 was evaluated under its fermentation in three different liquid media (YM, Q6 1/2, and ZM 1/2) and one solid medium (BRFT). This strain was identified based on sequence data of the internal transcribed spacer (ITS) regions and a fragment of the second largest subunit of the DNA-directed RNA polymerase II (*rpb2*) gene, which showed 100 and 99.81% nucleotide similarity with the type strain of *Amesia atrobrunnea* CBS 379.66 (GenBank accession numbers JX280771 and KX976798), respectively. The obtained crude extracts were analyzed by ultrahigh-performance liquid chromatography coupled to a diode array detector and high-resolution electrospray ionization mass spectrometry (UHPLC-DAD-HR-ESI-MS) and evaluated for their antimicrobial activity against a set of different bacterial and fungal pathogens. Distinct profiles of secondary metabolites were observed in the evaluated media. Dereplication of the major metabolites produced in each medium allowed us to identify the production of chetracin-like epithiodiketopiperazines by this fungus, including some that appeared to be unknown according to the accurate molecular formula prediction. One of these compounds was represented by a peak eluting at 6.86 min (**1**, *m*/*z* = 665.1484 Da). This compound was found to be produced in a higher quantity in the ZM 1/2 medium than in the other media. Consequently, we embarked on the targeted isolation of these metabolites after the scale-up fermentation of *A. atrobrunnea*.

Neochetracin (**1**) was isolated from the mycelial extract obtained after the 4-L cultivation of *A. atrobrunnea* FMR 19325 in the ZM 1/2 medium. Compound **1** was obtained as a white solid powder. Its molecular formula was established as C_30_H_28_N_6_O_8_S_2_ based on HR-ESI-MS experiments, which revealed a protonated molecular ion peak at *m*/*z* 665.1490 [M + H]^+^ (calculated to be 665.1483) and a sodium adduct at *m*/*z* 687.1310 [M + Na]^+^ (calculated to be 687.1302), indicating 20 degrees of unsaturation. To the best of our knowledge, this molecular formula was unprecedentedly reported in the literature for the epipolythiodioxopiperazine class; however, other reported derivatives featured a multiple of two sulfur atoms corresponding to four, six, and eight sulfur atoms as in melinacidin IV^8,9^ and chetracins C^10^ and A,^11,12^ respectively. Interestingly, all other derivatives possessing even numbers of sulfur atoms turned out to be symmetric dimers consisting of two identical monomers; therefore, their ^1^H and ^13^C NMR spectra displayed electromagnetically equivalent peaks corresponding to only one monomer. The ^13^C NMR and HSQC spectra of **1** (Table 1) showed the presence of 30 different carbon signals differentiated into four carbonyl groups (δ_C_ 167.0, 164.0, 163.9, and 162.4), four unprotonated sp^2^ carbon atoms (δ_C_ 151.8, 150.5, 128.1, and 126.4), and eight tertiary sp^2^ carbon atoms (δ_C_ 130.6, 130.0, 126.8, 124.5, 119.8, 119.5, 111.3, and 110.8) that account for 10 degrees of unsaturation. In addition, they also revealed the presence of five unprotonated sp^3^ carbon atoms (δ_C_ 86.0, 79.3, 77.6, 59.6, and 58.7), five tertiary sp^3^ carbon atoms (δ_C_ 81.7, 79.4, 77.8, 76.5, and 68.4), two secondary sp^3^ carbon atoms (δ_C_ 63.5 and 60.3), and two primary carbon atoms (δ_C_ 33.1 and 27.8).

**Table 1:** ^1^H and ^13^C NMR Data of Neochetracin (**1**)

	**1**	
pos.	δ_C_ type^a^^,^^c^	δ_H_ (multi, J [Hz])^b^
1	164.0, CO	
3	79.3, C	
4	162.4, CO	
5a	81.7, CH	5.14 s
6a	151.8, C	
7	110.8, CH	6.67 d (7.8)
8	130.6, CH	6.98 dt (7.6, 1.2)
9	119.5, CH	6.62 t (overlapped)
10	126.8, CH	7.32 d (7.7)
10a	128.1, C	
10b	58.7, C	
11	79.4, CH	7.44 d (3.0)
11a	77.6, C	
12	27.8, CH_3_	3.07 s
13	60.3, CH_2_	α 4.33 d (12.6)
		β 4.42 d (12.6)
11-OH		6.44 br s
13-OH		4.39 br s
1′	163.9, CO	
3′	68.4, CH	4.25 br s
4′	167.0, CO	
5a′	76.5, CH	4.37 br d (1.4)
6a′	150.5, C	
7′	111.3, CH	6.60 d (7.6)
8′	130.0, CH	6.98 dt (7.6, 1.2)
9′	119.8, CH	6.61 t (7.7)
10′	124.5, CH	7.59 dd (7.5, 1.3)
10a′	126.4, C	
10b′	59.6, C	
11′	77.8, CH	6.59 d (5.5)
11a′	86.0, C	
12′	33.1, CH_3_	3.10 s
13′	63.5, CH_2_	α 4.15 dd (12.6, 6.8)
		β 4.26 m
6′-NH		4.84 br d (2.4)
11′-OH		6.24 br d (4.8)
13′-OH		4.75 br s

a Measured in acetone-*d*_6_ at 175 MHz.

b Measured in acetone-*d*_6_ at 700 MHz.

c Assigned based on HMBC and HSQC spectra.

**Figure 1:**
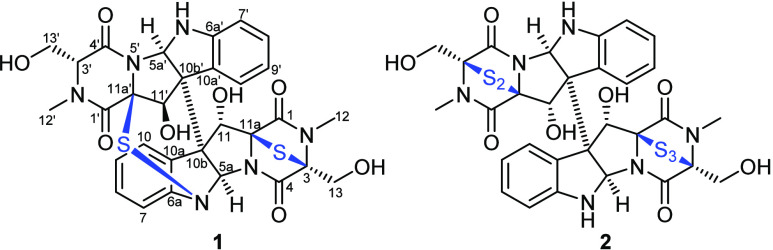
Structures of neochetracin (**1**) and chetracin B (**2**).

**Figure 2:**
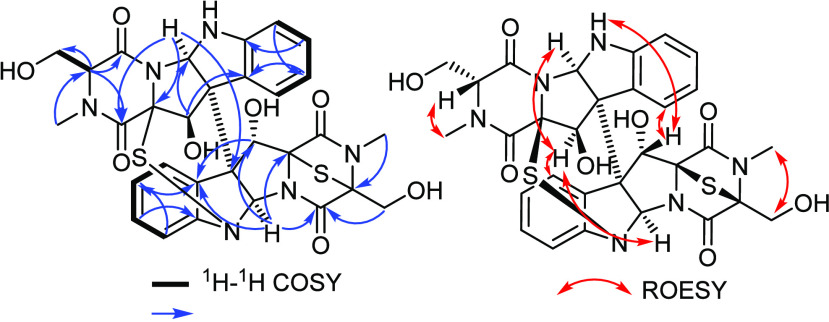
Key ^1^H–^1^H COSY, HMBC, and ROESY correlations of **1**.

Based on the obtained results, compound **1** was suggested to be an asymmetric epidithiodiketopiperazine derivative featuring a chemical scaffold similar to those of chetracins (Figure 1).^10−12^ The ^1^H NMR, ^1^H–^1^H COSY, and HSQC spectra (Table 1 and Figures 2 and S7) revealed the presence of two different characteristic olefinic spin systems (δ_H_ 6.60–7.59; δ_C_ 110.8–130.6) indicating the existence of two 1,2-disubstituted aromatic rings each ascribed to one monomer. The ^1^H–^1^H COSY and HSQC spectra of **1** (Figures 2 and S7) revealed three additional long-range spin systems as follow: 1) from a deshielded methine proton at δ_H_ 6.59 (d, *J* = 5.5 Hz, H-11′; δ_C_ 77.8) to an aliphatic methine at δ_H_ 4.37 (br d, *J* = 1.4 Hz, H-5a′; δ_C_ 76.5) extending to two exchangeable protons at δ_H_ 4.84 (br d, *J* = 2.4 Hz, 6′-NH) and 6.24 (br d, *J* = 4.8 Hz, 11′-OH); 2) between two aliphatic methine protons at δ_H_ 5.14 (s, H-5a; δ_C_ 81.7) and 7.44 (br s, H-11; δ_C_ 79.4) extending over one exchangeable proton at δ_H_ 6.44 (br s, 11-OH) with the absence of the 6-NH proton; and 3) from an aliphatic methine proton at δ_H_ 4.25 (br s, H-3′; δ_C_ 68.4) and diastereotopic hydroxymethylene protons at δ_H_ 4.15/4.26 (H_2_-13′) that were directly correlated by the HSQC spectrum to the same carbon resonance at δ_C_ 63.5 (C-13′). To further confirm the position of the latter spin system, the HMBC spectrum (Figure 2, Table S1, Figure S6) was recorded, and it revealed clear key correlations from H-3′ to two carbonyl carbon atoms of one diketopiperazine moiety at δ_C_ 167.0 (C-4′) and δ_C_ 163.9 (C-1′). This notation indicated that one of the two sulfur atoms could not be bridged between C-3′ and C-11a′ as in other epipolythiodiketopiperazine derivatives.^8−13^ The HMBC spectrum also revealed key correlations from an aliphatic methine at H-5a′ to a carbonyl carbon at δ_C_ 163.9 (C-1′), δ_C_ 126.4 (C-10a′), δ_C_ 86.0 (C-11a′), δ_C_ 59.6 (C-10b′), and δ_C_ 58.7 (C-10b), confirming their assignment to the same monomer of compound **1** and to be bound to the second via a C-10b-C10b′ linkage as for other chetracins.^8−12^ Similarly, the HMBC spectrum unraveled key correlations from an aliphatic methine singlet H-5a to a carbonyl carbon at δ_C_ 162.4 (C-4), 128.1 (C-10a), 79.4 (C-11), 77.6 (C-11a), and 58.7 (C-10b), suggesting their presence on the other monomer of **1**. Based on the molecular formula of **1** and by comparing the obtained results with reported spectral data of chetracins,^8−12^ it was suggested that the second sulfur atom bridges between N-6 of one monomer and C-11a of the other monomer, illustrating a unique C-11a′-S-N-6 (or C-11a-S-N-6′ in an inverted conformer) cross-linkage that is unprecedentedly featured in a natural product. According to the aforementioned data, compound **1** was deduced to be a new epidithiodiketopiperazine derivative whose structure is as depicted in Figure 1 and was given the trivial name neochetracin. The relative configuration of **1** was determined based on the key ROE correlations noticed in the ROESY spectrum (Figure 2, Table S1, and Figure S8). The ROESY spectrum displayed key correlations from H-5a to H-10, H_2_-13′, and H-11′ whereas the latter in turn revealed key ROE correlations to H-5a′ and H-10′, indicating that H-5a, H-5a′, H-11′, and H_2_-13′ are cofacial and opposite to H-11. Further key ROE correlations (Figure 2) were identified among H-11/H-10′, H-3′/H_3_-12′, and H_3_-12/H_2_-13, hence suggesting the relative configuration to be (3S*,5aS*,10bS*,11S*,11aS*,3′R*,5a′R*,10b′S*,11′R*,11a′S*).

The absolute configuration of **1** was not possible to determine by a comparison of its experimental and calculated ECD spectra due to its high number of chiral centers reflected in a tremendous number of conformers to be assessed that made the calculations not conclusive, and these findings came in accordance with a previous report on chetracins.^10^

In addition, compound **2** was also obtained as a solid brown powder, and its molecular formula was established to be C_30_H_28_N_6_O_8_S_5_ based on HR-ESI-MS that exhibited a protonated molecular ion peak at *m*/*z* 761.0643 [M + H]^+^ (calculated to be 761.0645) and a sodium adduct at *m*/*z* 783.0464 [M + Na]^+^ (calculated to be 783.0464) indicating 20 degrees of unsaturation. A literature search of **2** and a comparison of its ^1^H and ^13^C NMR data (Table S2) confirmed its identity as chetracin B, previously reported from the Antarctic psychrophilic fungus *Oidiodendron truncatum*.^10^

Compounds **1** and **2** were evaluated for their antimicrobial activity against a panel of fungal and bacterial pathogens. However, none of them revealed significant antifungal activity. Chetracin B (**2**) exhibited a higher activity against Gram-positive rather than Gram-negative bacteria, except for *Chromobacterium violaceum* with an MIC value of 8.3 μg/mL (Table 2). In contrast, neochetracin (**1**) was less active against the tested bacteria but still retained inhibition against *Bacillus subtilis* at 0.52 μg/mL, which was more potent than the positive control, oxytetracycline (MIC = 8.3 μg/mL). Both compounds **1** and **2** were assessed for their cytotoxic activities against seven different cell lines. The obtained results (Table 3) showed that both **1** and **2** featured remarkable pancytotoxic activities against all tested cell lines with IC_50_ values in the nanomolar range comparable to and sometimes more potent than epothilone B used in our experiments as a positive control. It notes that both compounds generally exhibited higher cytotoxicity against the mouse fibroblast cell line (L929) compared to the six evaluated cancer cell lines, which might be significant for the potential application of these compounds in cancer therapeutics.

**Table 2:** Minimum Inhibitory Concentration (MIC, μg/mL) of **1** and **2**

Test organism	**1**	**2**	Control
*Acinetobacter baumanii*	f	16.6	0.26^a^
*Bacillus subtilis*	0.52	0.03	8.3^b^
*Candida albicans*	f	f	8.3^c^
*Chromobacterium violaceum*	f	f	0.42^b^
*Escherichia coli*	f	66.6	1.7^b^
*Mucor hiemalis*	f	f	8.3^c^
*Mycobacterium smegmatis*	f	f	1.7^e^
*Pseudomonas aeruginosa*	f	66.6	0.21^d^
*Rhodotorula glutinis*	f	f	4.2^c^
*Schizosaccharomyces pombe*	f	f	8.3^c^
*Staphylococcus aureus*	33.3	8.3	0.42^b^
*Wickerhamomyces anomalus*	f	f	8.3^c^

a Ciprobay.

b Oxytetracycline.

c Nystatin.

d Gentamycin.

e Kanamycin.

f No inhibition observed under test conditions.

**Table 3:** Cytotoxicity (IC_50_ in nM) of **1** and **2**

Cell line	**1**	**2**	Epothilone B
L929	0.72	0.01	0.65
KB3.1	2.56	0.033	0.17
A549	3.46	0.06	0.53
A431	0.71	0.004	0.07
PC-3	3.77	0.10	0.09
MCF-7	0.39	0.03	0.07
SKOV-3	3.46	0.05	0.09

Epithiodiketopiperazines and their representatives have been investigated due to their broad spectrum of cytotoxic activities. For instance, the related compound chaetocin has shown a wide spectrum of antitumor activities *in vivo* with IC_50_ values in the nanomolar range.^14^ Similarly, chetracin B (**2**) has been previously indicated to act as a heat shock protein 90 (Hsp90) inhibitor by its binding to the C-terminal.^13^ These results have supported extensive research of this class of molecules, since Hsp90 has been validated to be a crucial target in cancer treatment. However, no structure–activity relationships (SAR) have been established for the chetracin-like epipolythiodiketopiperazines despite the interesting biological properties they harbor. Herein, both isolated compounds presented strong cytotoxic effects against all seven tested cell lines (Table 3). To fully understand the function of the chemical features found in these molecules and how they influence the exerted biological activities, future studies should be planned to systematically establish structure–activity relationships.

## 3 Conclusions

To the best of our knowledge, this study represents the first report of secondary metabolites produced by the fungus *Amesia atrobrunnea*, demonstrating its capacity to produce chetracin-like ETPs even featuring an unprecedented C-11a′-S-N cross-linkage within this compound class. Our findings highlight the diversity for this class of compounds and expand our knowledge on the biosynthetic diversity of ETPs. While chetracin derivatives are connected with a broad spectrum of biological activities, their potential use is limited by inherent toxicity to both healthy and cancer cell lines. Their future applicability will require advancing our molecular understanding of their biological function and expanding the synthetic strategies to optimize this chemical scaffold.

## 4 Materials and Methods

### 4.1 General Experimental Procedure

Optical rotations were determined using an MCP 150 polarimeter at 20 °C (Anton-Paar Opto Tec GmbH, Seelze, Germany). Ultraviolet–visible (UV/vis) spectra were obtained using a UV–vis spectrophotometer UV-2450 (Shimadzu, Kyoto, Japan). NMR spectra were recorded with an Avance III 700 spectrometer (Bruker, Billerica, MA, USA; ^1^H NMR: 700 MHz and ^13^C NMR: 175 MHz) and an Avance III 500 spectrometer (Bruker, Billerica, MA, USA, ^1^H NMR: 500 MHz, and ^13^C NMR: 125 MHz) for compounds dissolved in deuterated acetone-*d*_6_ or deuterated DMSO-*d*_6_.

High-resolution electrospray ionization mass spectra (HR-ESI-MS) were acquired with an Agilent 1200 Infinity Series HPLC-UV system (Agilent Technologies, Santa Clara, CA, USA) utilizing a C_18_ Acquity UPLC BEH column [2.1 × 50 mm; 1.7 μm: Waters, Milford, MA, USA; solvent A (deionized water (H_2_O) + 0.1% formic acid), solvent B (acetonitrile (MeCN) + 0.1% formic acid), gradient (5% B for 0.5 min increasing to 100% B in 19.5 min, holding 100% B for 5 min, flow rate of 0.6 mL min^–1^, UV/vis detection 190–600 nm] connected to a time-of-flight mass spectrometer (ESI-TOF-MS, maXis, Bruker, Billerica, MA, USA; scan range 100–2500 *m*/*z*, rate 2 Hz, capillary voltage 4500 V, dry temperature 200 °C). Molecular formulas of the detected compounds were calculated using the Smart Formula algorithm of the Compass Data Analysis software (Bruker, version 6.1).

### 4.2 Isolation and Identification of the Fungus

Soil samples from the rhizosphere of *Vitis vinifera* were collected in a Celler Clos Mogador in Gratallops, Spain. For the isolation of the fungus, we followed a previously described procedure to activate dormant spores in soil using 2% (v/v) phenol.^7^ The fungus was first classified as a member of the family Chaetomiaceae based on its morphological characteristics (i.e., ascomata with hairs surrounding the ostiole, fasciculate, and evanescent asci along with one-celled, smooth-walled, and olivaceous brown ascospores with a germ pore in one end). Subsequently, this was identified to the species level based on sequence data. DNA of the isolate was extracted and purified directly from colonies according to the Fast DNA Kit protocol (MP Biomedicals, Solon, Ohio). The amplification of the ITS and *rpb2* was performed according to White et al. (ITS; primers ITS5 and ITS4),^15^ and Miller and Huhndorf (*rpb2*; primers RPB2AM-1bf and RPB2AM-7).^16^ PCR products were purified and sequenced at Macrogen Europe (Amsterdam, The Netherlands) with a 3730XL DNA analyzer (Applied Biosystems). Consensus sequences were obtained using Geneious 7.1.9.^17^ and compared with sequences from the GenBank (www.ncbi.nlm.nih.gov) database with a BLAST search to achieve the identification at the species level.

The fungus is maintained at the collection of the Facultad de Medicina y Ciencias de la Salud, University Rovira i Virgili, Reus, Spain (FMR), and the sequences generated in this study were deposited in GenBank (OQ743414 and QQ753884 for ITS and *rpb2* loci, respectively).

### 4.3 Fermentation, Extraction, and Isolation

To evaluate the secondary metabolite production of *Amesia atrobrunnea* FMR 19325, three different liquid media (YM 6.3, ZM 1/2, and Q6 1/2) and one solid medium (BRFT) were used.^7^ The fungus was initially grown on yeast malt agar (YM agar) at 23 °C. Subsequently, the mycelia were cut into small pieces using a cork borer (1 cm × 1 cm), with eight pieces placed into each 500 mL Erlenmeyer flask containing 200 mL of each liquid medium. These cultures were incubated at 23 °C and 140 rpm in darkness until 3 days after glucose depletion. Additionally, for solid cultures, an extra 500 mL Erlenmeyer flask containing 200 mL of YM broth was incubated under the same conditions. After 7 days, 6 mL of this seed culture was transferred to a 500 mL Erlenmeyer flask containing the BRFT medium. The solid culture was incubated for 15 days at 23 °C in darkness under static conditions.

To extract the secondary metabolites from liquid cultures, the mycelia were first separated from the supernatant through filtration. The supernatant was extracted with an equal volume of ethyl acetate (EtOAc) in a separatory funnel. The resulting EtOAc fraction was evaporated to dryness under vacuum at 40 °C. Meanwhile, the mycelia were sonicated in acetone using an ultrasonic bath for 30 min at 40 °C, and the obtained acetone fraction was separated from the mycelia by filtration throughout cellulose filter paper (MN 615 1/4 Ø 185 mm, Macherey-Nagel, Düren, Germany). The remaining mycelia went through another round of sonication/extraction, and both extracts were combined and evaporated to yield an aqueous residue, which was extracted in a manner similar to that of the supernatant. The solid cultures were extracted similarly to the mycelia obtained from liquid cultures until the evaporation of the EtOAc fraction. Afterward, the obtained extract was dissolved in methanol and partitioned with an equal volume of *n*-heptane in a separatory funnel. This step was repeated with the obtained methanol phase, which was then evaporated to dryness under a vacuum at 40 °C. The methanol fractions were finally combined and dried under a vacuum at 40 °C.

For the scaled-up cultivation, the fungus was grown in YM agar at 23 °C. Later, the mycelia were cut into small pieces using a cork borer (1 cm × 1 cm), and eight pieces were placed into each of the 20 × 500 mL Erlenmeyer flasks containing 200 mL of ZM 1/2 broth (4 L in total) and incubated at 23 °C and 140 rpm in darkness until 3 days after glucose depletion. Consequently, the cultures followed the extraction procedure described above to afford 1.85 g and 558 mg of supernatant and mycelial extract, respectively.

The mycelial extract was separated using a PLC 2250 preparative HPLC system (Gilson, Middleton, WI, USA) with a Gemini C_18_ (250 × 50 mm, 10 μm, Phenomenex, Torrance, CA, USA) as the stationary phase and the following conditions as the mobile phase: solvent A [deionized water (H_2_O) + 0.1% formic acid], solvent B [acetonitrile (MeCN) + 0.1% formic acid], 40 mL/min flow, and a 15 mL collected fraction volume. The following gradient elution was applied: increasing from 5% B to 35% B in 15 min, from 35% B to 56% B in 45 min, and finally from 56% B to 100% B in 10 min. Ten fractions (MF1-MF10) were collected, from which fraction MF9 (114 mg) was further purified in several runs with an Agilent Technologies 1200 Infinity Series semipreparative reverse-phase HPLC using an XBridge BEH C_18_ column (250 × 10 mm, 5 μm, Waters)^18^ as the stationary phase and the following conditions as the mobile phase: solvent A [deionized water (H_2_O) + 0.1% formic acid], solvent B [acetonitrile (MeCN) + 0.1% formic acid], and 3 mL/min flow. The following gradient elution was applied: increasing from 35 to 50% B in 60 min. This afforded compounds **1** (0.6 mg, *t*_R_ = 25.4 min) and **2** (1.3 mg, *t*_R_ = 26.9 min).

#### 4.3.1 Neochetracin (**1**)

Solid white powder. [α]_D_^25^ +3.26 (*c* 0.1, CDCl_3_). UV/vis (MeOH): λ_max_ (log ε) = 293.5 (4.07), 241.0 (4.45) nm. NMR data (^1^H NMR: 700 MHz; ^13^C NMR: 175 MHz in acetone-*d*_6_), see Table 1. HR-(+)ESI-MS: *m*/*z* 647.1386 [M – H_2_O + H]^+^ (calcd. 647.1377 for C_30_H_27_N_6_O_7_S_2_^+^), 665.1490 [M + H]^+^ (calcd. 665.1483 for C_30_H_29_N_6_O_8_S_2_^+^), 687.1310 [M + Na]^+^ (calcd. 687.1302 for C_30_H_28_N_6_NaO_8_S_2_^+^); *t*_R_ = 6.86 min (LC-ESI-MS).

#### 4.3.2 Chetracin B (**2**)

Solid brown powder. [α]_D_^25^ +3.65 (*c* 0.1, CDCl_3_). UV/vis (MeOH): λ_max_ = 297.5 (3.87), 240.5 (4.36), 200 (3.96) nm. NMR data (^1^H NMR: 500 MHz; ^13^C NMR: 125 MHz in DMSO-*d*_6_); see Table S2, which is comparable to the reported literature.^10^ HR-(+)ESI-MS: *m*/*z* 761.0643 [M + H]^+^ (calcd. 761.0645 for C_30_H_29_N_6_O_8_S_5_^+^), 783.0464 [M + Na]^+^ (calcd. 783.0464 for C_30_H_28_N_6_NaO_8_S_5_^+^). *t*_R_ = 8.84 min (LC-ESI-MS).

### 4.4 Antimicrobial Assay

The antifungal and antibacterial activities (minimum inhibition concentration, MIC, in μg/mL) of all extracts and isolated compounds were determined in serial dilution assays as previously described.^7^ An array of clinically relevant microorganisms including bacteria, namely, *Staphylococcus aureus* (DSM 346), *Acinetobacter baumanii* (DSM 30008), *Bacillus subtilis* (DSM 10), *Escherichia coli* (DSM 1116), *Pseudomonas aeruginosa* (PA14), *Chromobacterium violaceum* (DSM 30191), and *Mycobacterium smegmatis* (ATCC 700084) and fungi such as *Mucor hiemalis* (DSM 2656), *Candida albicans* (DSM 1665), *Rhodotorula glutinis* (DSM 10134), *Schizosaccharomyces pombe* (DSM 70572), and *Pichia anomala* (DSM 6766), were used.

### 4.5 Cytotoxicity Assay

The *in vitro* cytotoxicity (IC_50_) assessments were carried out on the isolated compounds based on the MTT (3-(4,5-dimethylthiayol-2-yl)-2,5-diphenyltetrazolium bromide) assay in 96-well plates using the cell lines L929 (mouse fibroblasts), KB3.1 (human endocervical adenocarcinoma), A431 (human squamous carcinoma), A549 (human lung carcinoma), PC-3 (human prostate adenocarcinoma), and MCF-7 (human breast adenocarcinoma) in accordance with our previously established methods.^7^

## Supporting Information Available

All 1D (^1^H/^13^C) and 2D (^1^H–^1^H COSY, HMBC, HSQC, and ROESY) spectra in addition to all HPLC-ESI-MS and HR-ESI-MS of neochetracin (**1**) and chetracin B (**2**) (PDF)
