# Supplementary material for: Neochetracin: An Unusual Chetracin-Type Epithiodiketopiperazine Derivative Produced by the Fungus Amesia atrobrunnea
Source: ACS Omega. 2024 May 17;9(22):24009–14. doi: 10.1021/acsomega.4c02424 (PMC11154914; doi:10.1021/acsomega.4c02424)
Supplement: Supplementary file 1 — ao4c02424_si_001.pdf [file ao4c02424_si_001.pdf]

Supporting Information for:

Neochetracin: An unusual chetracin-type epithiodiketopiperazine derivative produced by the fungus *Amesia atrobrunnea*

Esteban Charria-Girón,[†,‡] Christina Sauer,[†] Dania García,[⊥] Sherif S. Ebada,[†,§]* and Yasmina Marin-Felix[†,‡]*

[†] Department Microbial Drugs, Helmholtz Centre for Infection Research, Inhoffenstraße 7, 38124 Braunschweig

[‡] Institute of Microbiology, Technische Universität Braunschweig, Spielmannstraße 7, 38106 Braunschweig, Germany

[⊥] Unitat de Micologia i Microbiologia Ambiental, Facultat de Medicina i Ciències de la Salut and IISPV, Universitat Rovira i Virgili, 43201 Reus, Spain

[§] Department of Pharmacognosy, Faculty of Pharmacy, Ain Shams University, 11566 Cairo, Egypt

* Corresponding authors: sherif.elsayed@helmholtz-hzi.de; sherif_elsayed@pharma.asu.edu.eg (S.S.E.); yasmina.marinfelix@helmholtz-hzi.de (Y.M.-F.); Tel.: +49-531-6181-4267; Fax +49-531-6181-9499

Contents of Supporting Information

#	Contents	Page
**1**	Table S1. 2D (^{1}H-^{1}H COSY, HMBC and ROESY) NMR data of **1**.	**S3**
**2**	Table S2. ^{1}H and ^{13}C NMR data of **2** and chetracin B.	**S4**
**3**	Figure S1. LRESIMS spectrum of **1**.	**S5**
**4**	Figure S2. HRESIMS spectrum of **1**.	**S5**
**5**	Figure S3. ^{1}H NMR spectrum of **1** in acetone-d_6 at 700 MHz.	**S6**
**6**	Figure S4. ^{13}C NMR spectrum of **1** in acetone-d_6 at 175 MHz.	**S7**
**7**	Figure S5. ^{1}H-^{1}H COSY spectrum of **1** in acetone-d_6 at 700 MHz.	**S8**
**8**	Figure S6. HMBC spectrum of **1** in acetone-d_6 at 700 MHz.	**S9**
**9**	Figure S7. HSQC spectrum of **1** in acetone-d_6 at 700 MHz.	**S10**
**10**	Figure S8. ROESY spectrum of **1** in acetone-d_6 at 700 MHz.	**S11**
**11**	Figure S9. LRESIMS spectrum of **2**.	**S12**
**12**	Figure S10. HRESIMS spectrum of **2**.	**S12**
**13**	Figure S11. ^{1}H NMR spectrum of **2** in chloroform-*d* at 500 MHz.	**S13**
**14**	Figure S12. ^{13}C NMR spectrum of **2** in chloroform-*d* at 125 MHz.	**S14**
**15**	Figure S13. ^{1}H-^{1}H COSY spectrum of **2** in chloroform-*d* at 500 MHz.	**S15**
**16**	Figure S14. HMBC spectrum of **2** in chloroform-*d* at 500 MHz.	**S16**
**17**	Figure S15. HSQC spectrum of **2** in chloroform-*d* at 500 MHz.	**S17**
**18**	Figure S16. ROESY spectrum of **2** in chloroform-*d* at 500 MHz.	**S18**

Table S1. 2D (^1H-^1H COSY, HMBC and ROESY) NMR data of **1**.

**pos.**	**^1H-^1H COSY[a]**	**HMBC[a]**	**ROESY[a]**
1			
3			
4			
5a		4, 10a, 11, 11a	H-7, H-10, H-11', 11-OH
6a			
7	H-8	9, 10a	
8	H-7, H-9	6a, 10	
9	H-8, H-10	7, 10w[b], 10a	
10	H-9	6a, 8, 10b	H-5a
10a			
10b			
11		5a, 6a, 10a, 11a, 10b'	H-10'
11a			
12		1, 13	H_2-13
13		3w[b], 4	H_3-12
1'			
3'	H_2-3'	1', 4', 12',13'	H_3-12'
4'			
5a'	6'-NH	10b , 4', 10a', 10b', 11a'	H-7', H-11', 6'-NH
6a'			
7'	H-8'	9', 10a'	
8'	H-7', H-9'	6a', 10'	
9'	H-8', H-10'	7', 10'w[b], 10a'	
10'	H-9'	6a', 8', 10b'	
10a'			
10b'			
11'		10b, 4'w[b], 5a',6a'w[b], 10a', 10b', 11a'	H-5a, H-10
11a'			
12'		1', 3'	H-3'
13'		3'	
11-O*H*		6a, 10a, 10b, 11a,	H-11, H-10'
13-O*H*			
6'-N*H*	H-5a'		H-5a'
11'-OH	H-11'	10a'w[b], 10b'w[b]	H-5a', H-11'
13'-O*H*			

[a] Measured in acetone-d_6 at 700 MHz. [b] "w" denotes weak correlations.

Table S2. ^{1}H and ^{13}C NMR data of compound (**2**) and chetracin B.[a]

	**2**		Chetracin B[a]	
pos.	δ_C,[b,f] type	δ_H[c] (multi, J [Hz])	δ_C,[d,f] type	δ_H[e] (multi, J [Hz])
1	169.0, CO		169.1, CO	
3	74.0, C		74.4, C	
4	165.3, CO		165.5, CO	
5a	84.5, CH	5.74 s	84.8, CH	5.74 s
6a	150.6, C		150.8, C	
7	111.0, CH	6.75 d (7.9)	111.2, CH	6.75 d (7.7)
8	131.0, CH	7.21 t (7.5)	131.0, CH	7.21 t (7.1, 7.7)
9	120.3, CH	6.82 t (overlapped)	120.3, CH	6.81 t (7.1)
10	129.2, CH	7.72 d (6.8)	128.6, CH	7.72 d (6.8)
10a	126.4, C		126.6, C	
10b	65.7, C		65.7, C	
11	81.7, CH	4.99 s (overlapped)	81.8, CH	4.99 s
11a	84.2, C		84.4, C	
12	27.4, CH_3	3.23 s	27.4, CH_3	3.23 s
13	62.2, CH_2	*α* 3.93 d (13.2)	62.2, CH_2	*α* 3.93 d (13.2)
		β 4.14 (overlapped)		*β* 4.16 (overlapped)
1’	166.3, CO		166.4, CO	
3’	75.2, C		75.5, C	
4’	162.6, CO		162.7, CO	
5a’	81.4, CH	5.64 s	81.5, CH	5.64 s
6a’	148.2, C		148.4, C	
7’	110.7, CH	6.67 dd (7.9, 4.9)	110.9, CH	6.66 dd (7.7)
8’	130.1, CH	7.16 t (8.1)	130.1, CH	7.15 t (7.1, 6.6)
9’	120.7, CH	6.84 t (overlapped)	120.7, CH	6.84 t (7.7, 6.6)
10’	128.1, CH	7.73 d (6.8)	128.6, CH	7.73 d (6.6)
10a’	129.0, C		128.9, C	
10b’	63.3, C		63.5, C	
11’	83.0, CH	5.07 s	83.0, CH	5.07 s
11a’	75.8, C		76.1, C	
12’	26.8, CH_3	3.09 s	27.0, CH_3	3.09 s
13’	60.2, CH_2	*α* 4.17 (overlapped)	60.2, CH_2	*α* 4.16 (overlapped)
		β 4.26 (overlapped)		*β* 4.25 (overlapped)

[a] Li et al., *J. Nat. Prod.* **2012**, *75*, 920−927; dx.doi.org/10.1021/np3000443
Measured in chloroform-*d* [b] at 125 MHz / [c] at 500 MHz / [d] at 100 MHz / [e] at 400 MHz.
[f] Assigned based on HMBC and HSQC spectra.

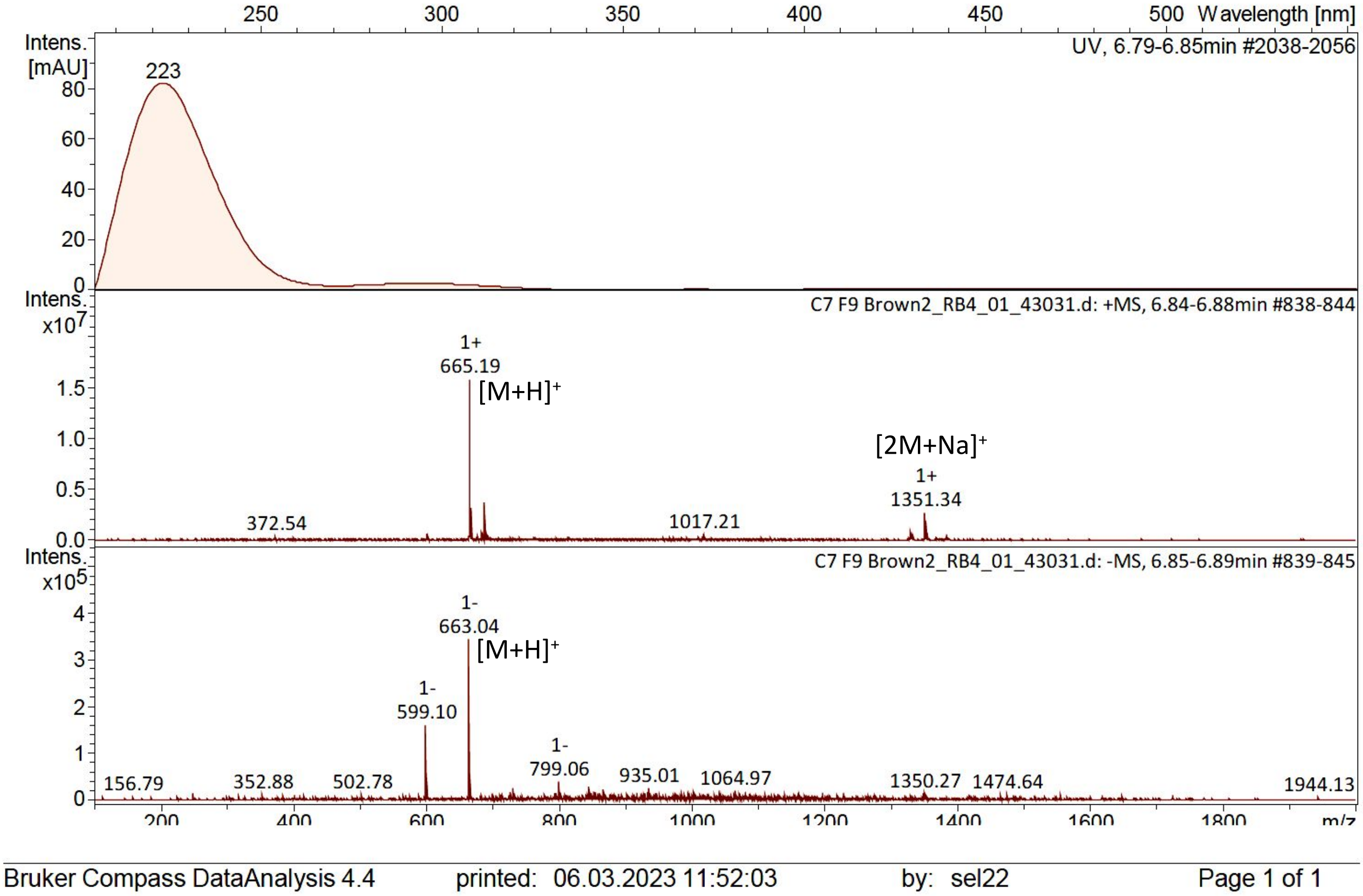

Figure S1. LRESIMS spectrum of **1**.

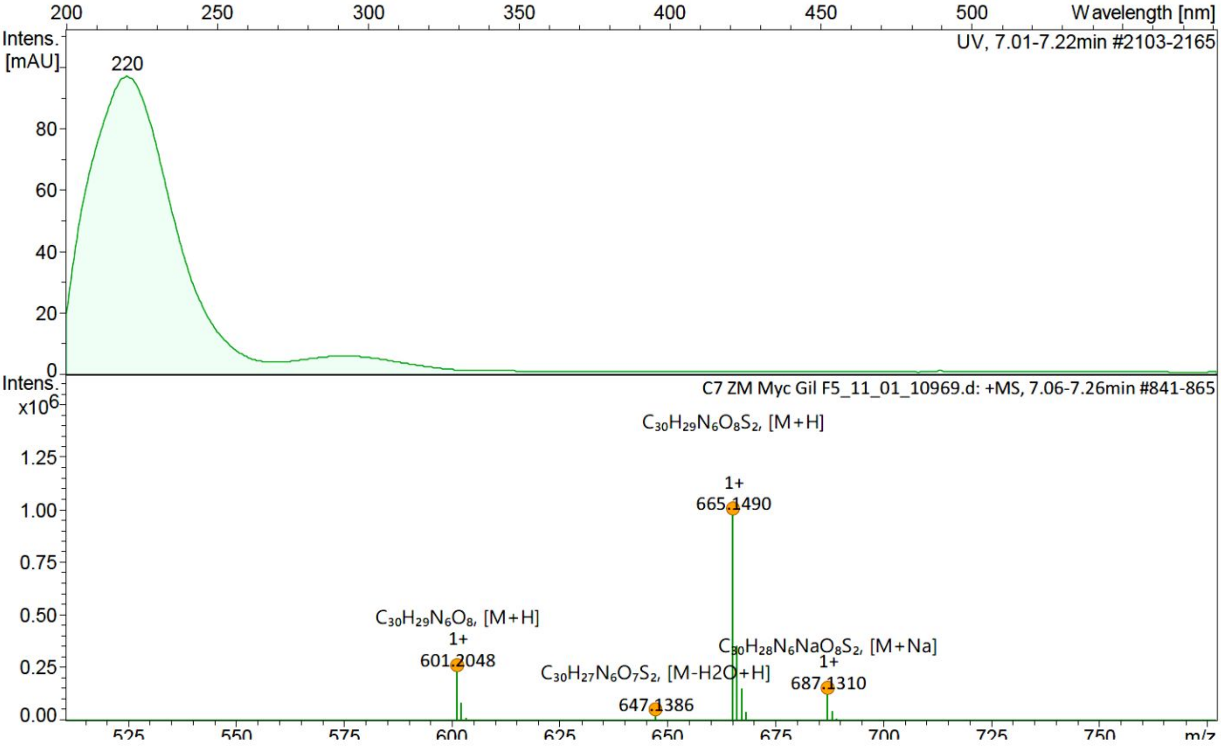

Figure S2. HPLC chromatogram and HRESIMS spectrum of **1**.

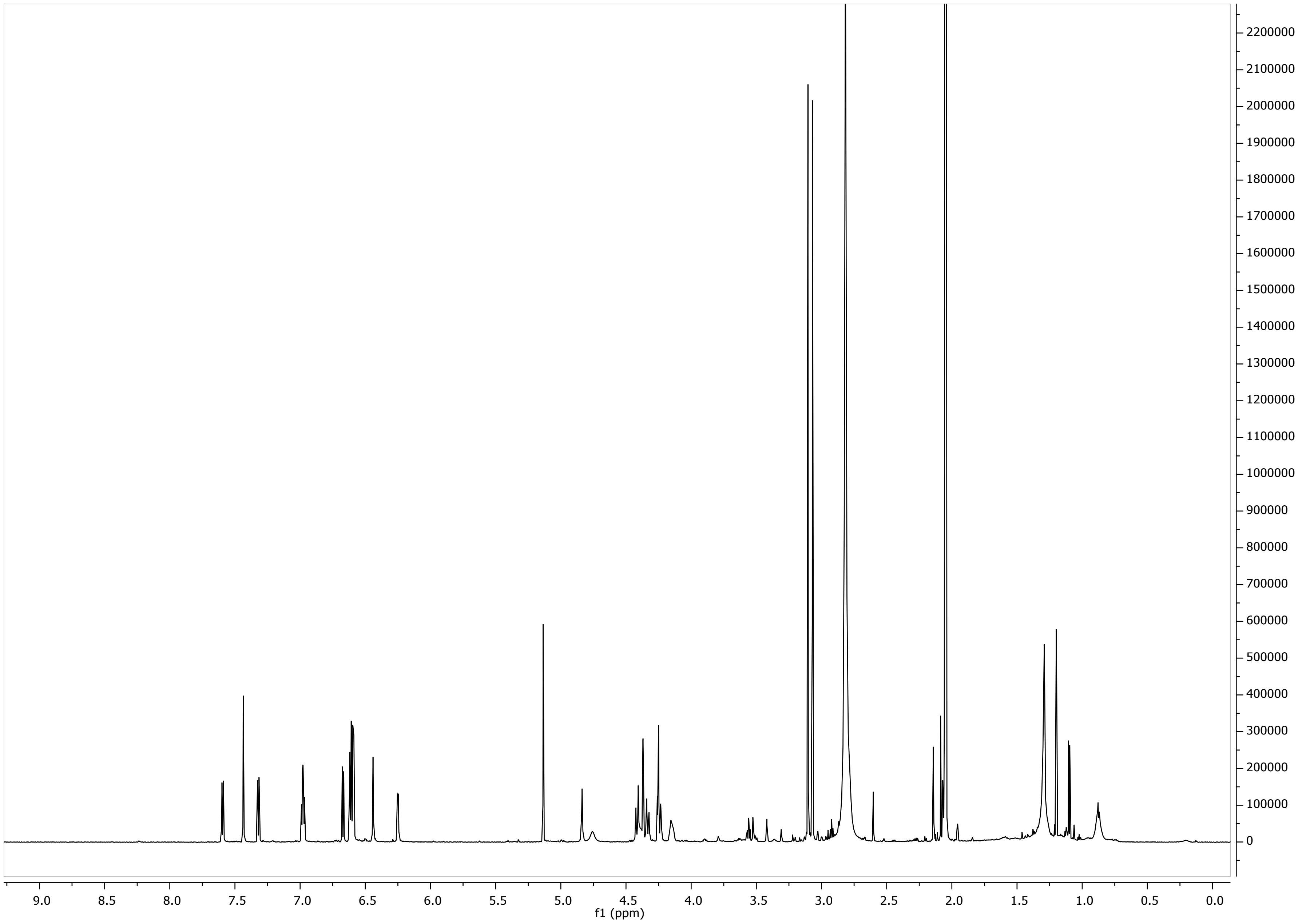

Figure S3. ^{1}H NMR spectrum of **1** in acetone-d_6 at 700 MHz.

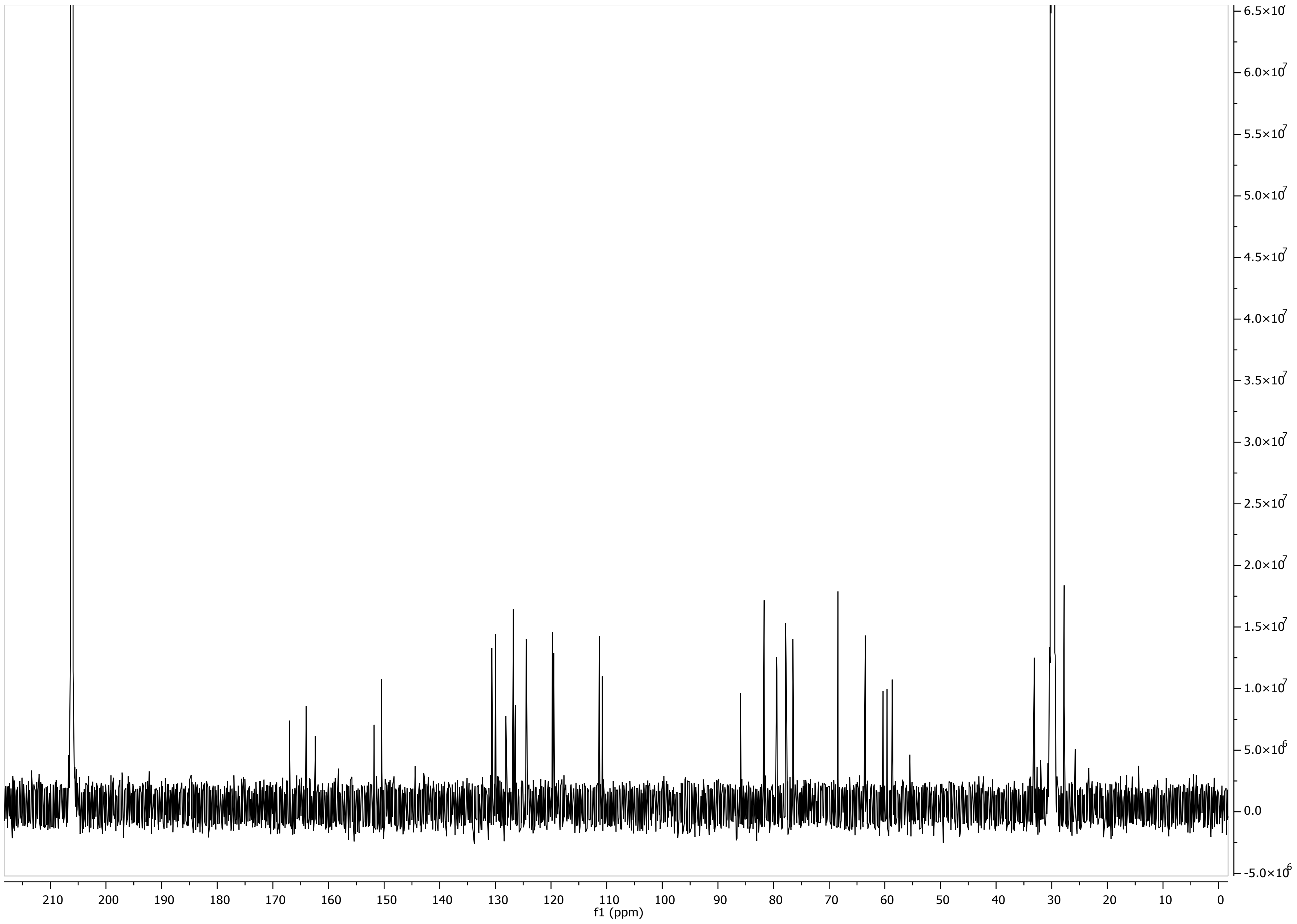

Figure S4. ^{13}C NMR spectrum of **1** in acetone-d_6 at 175 MHz.

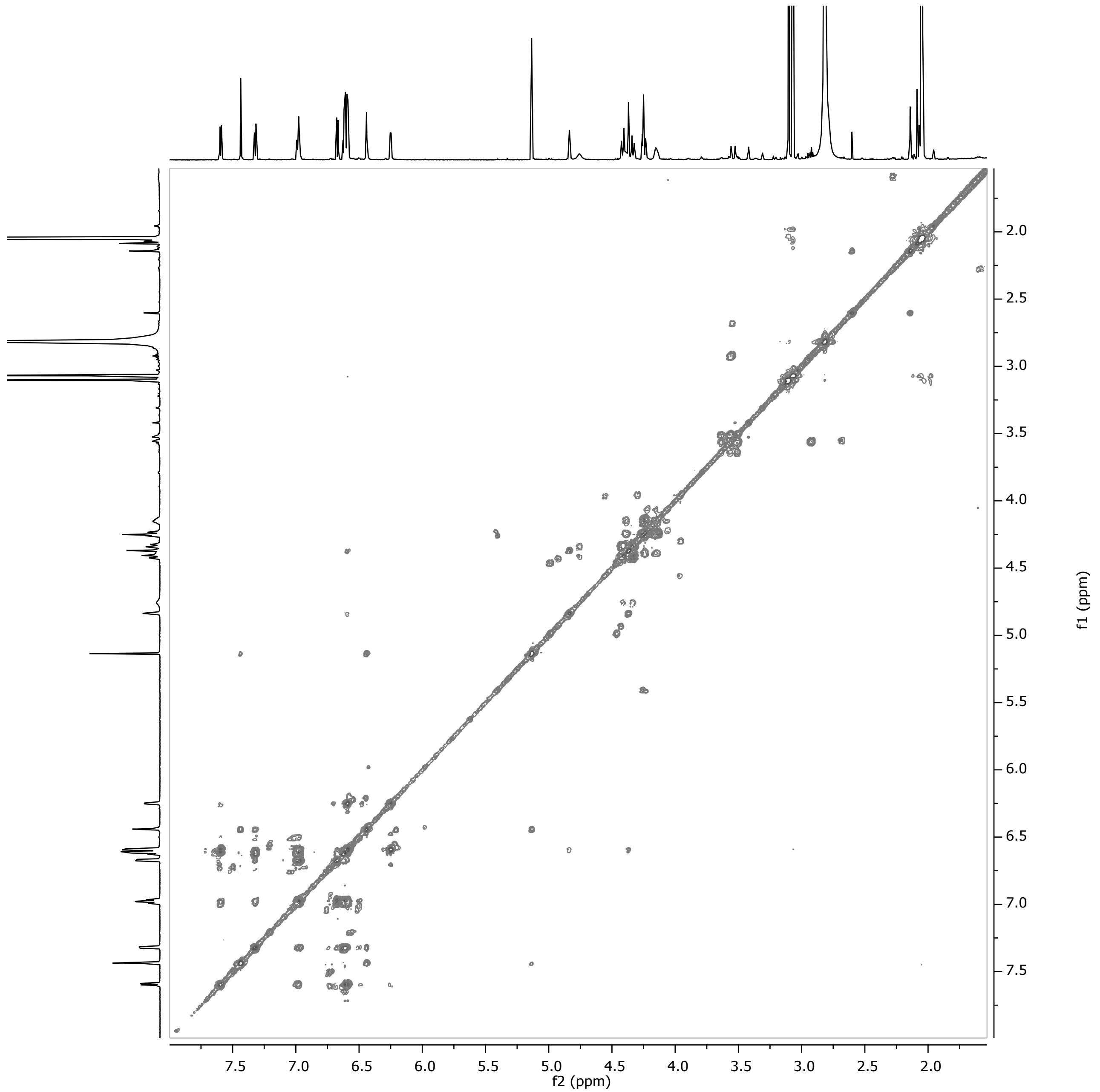

Figure S5. ^1H-^1H COSY spectrum of **1** in acetone-d_6 at 700 MHz.

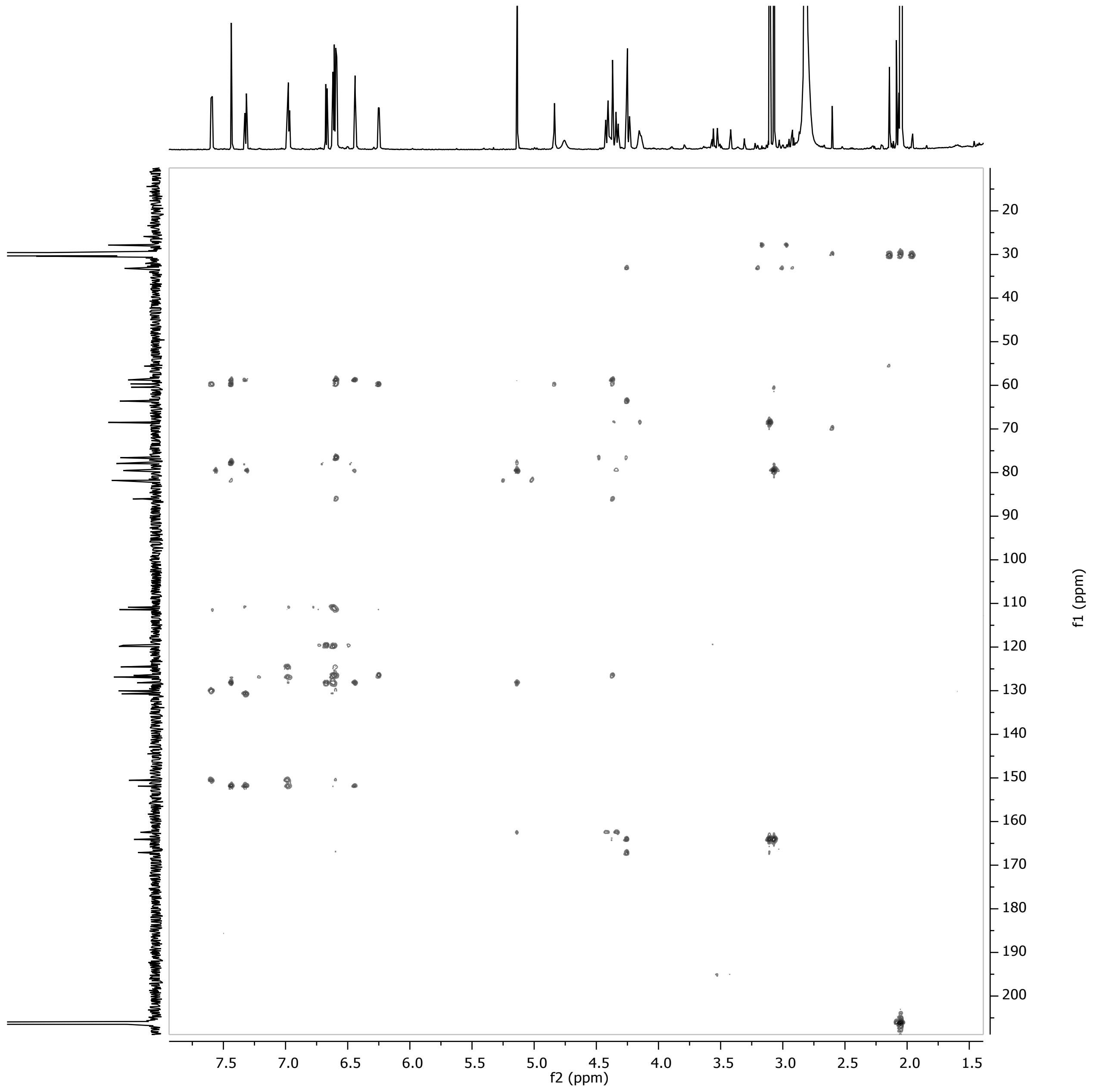

Figure S6. HMBC spectrum of **1** in acetone-d_6 at 700 MHz.

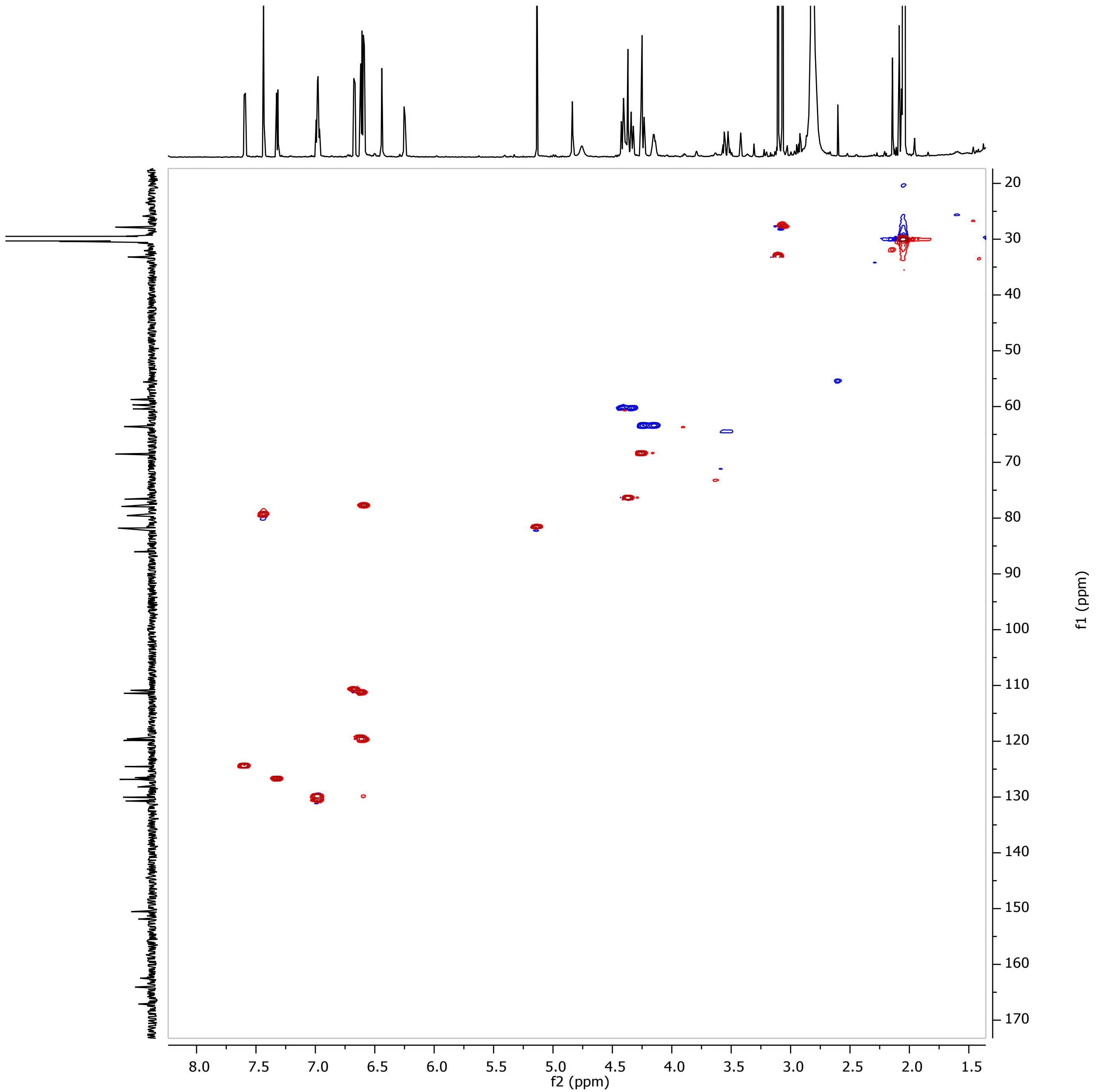

Figure S7. HSQC spectrum of **1** in acetone-d_6 at 700 MHz.

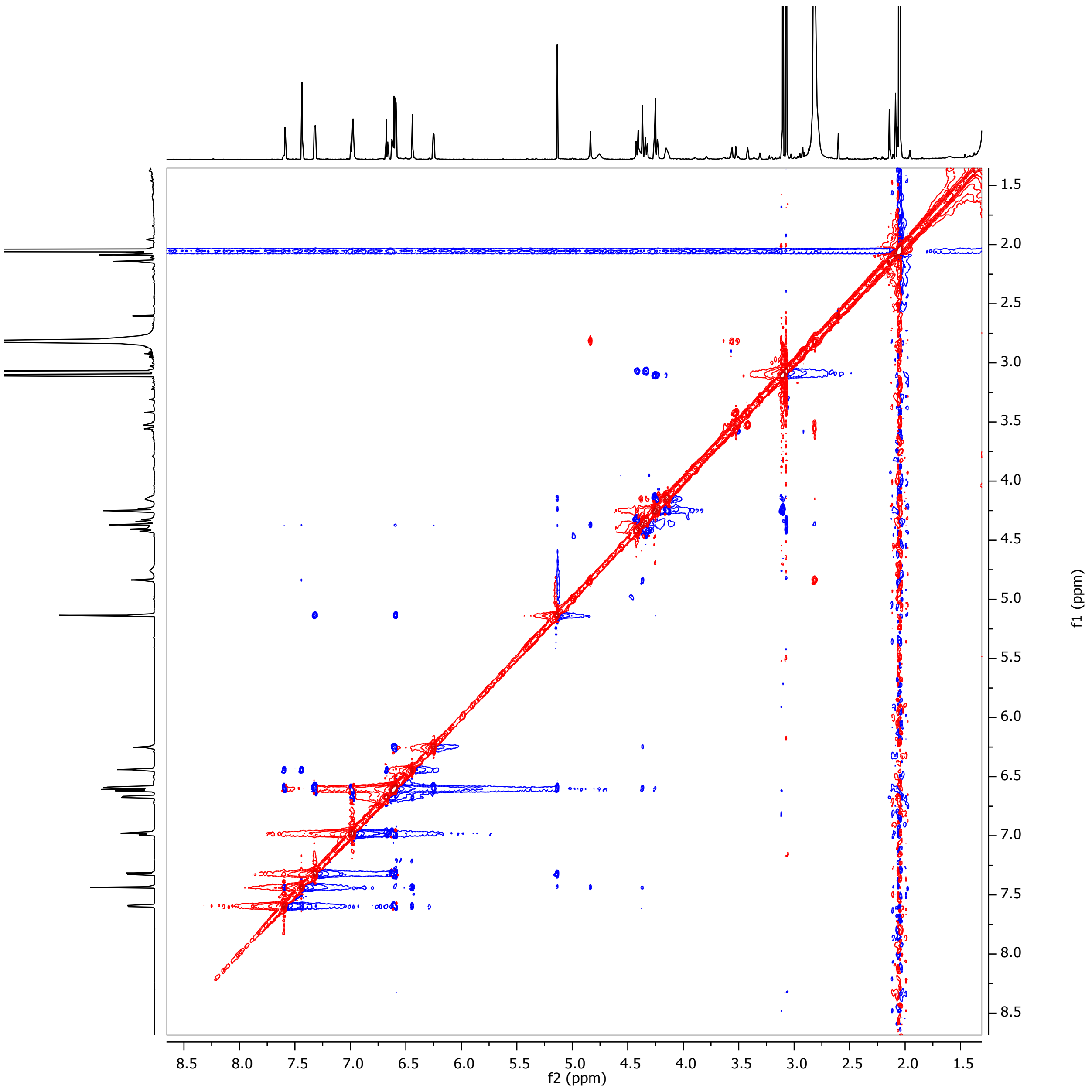

Figure S8. ROESY spectrum of **1** in acetone-d_6 at 700 MHz.

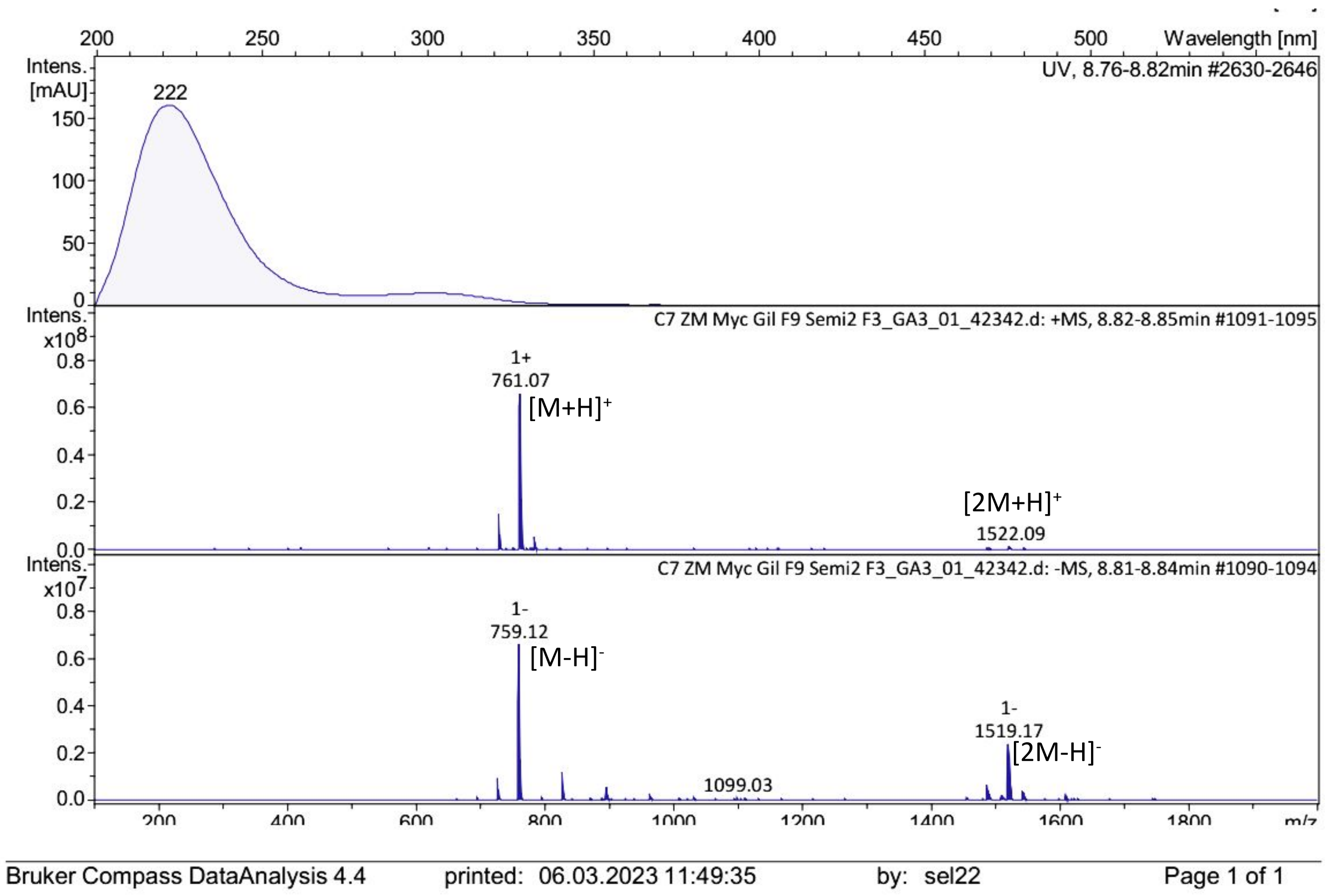

Figure S9. LRESIMS spectrum of **2**.

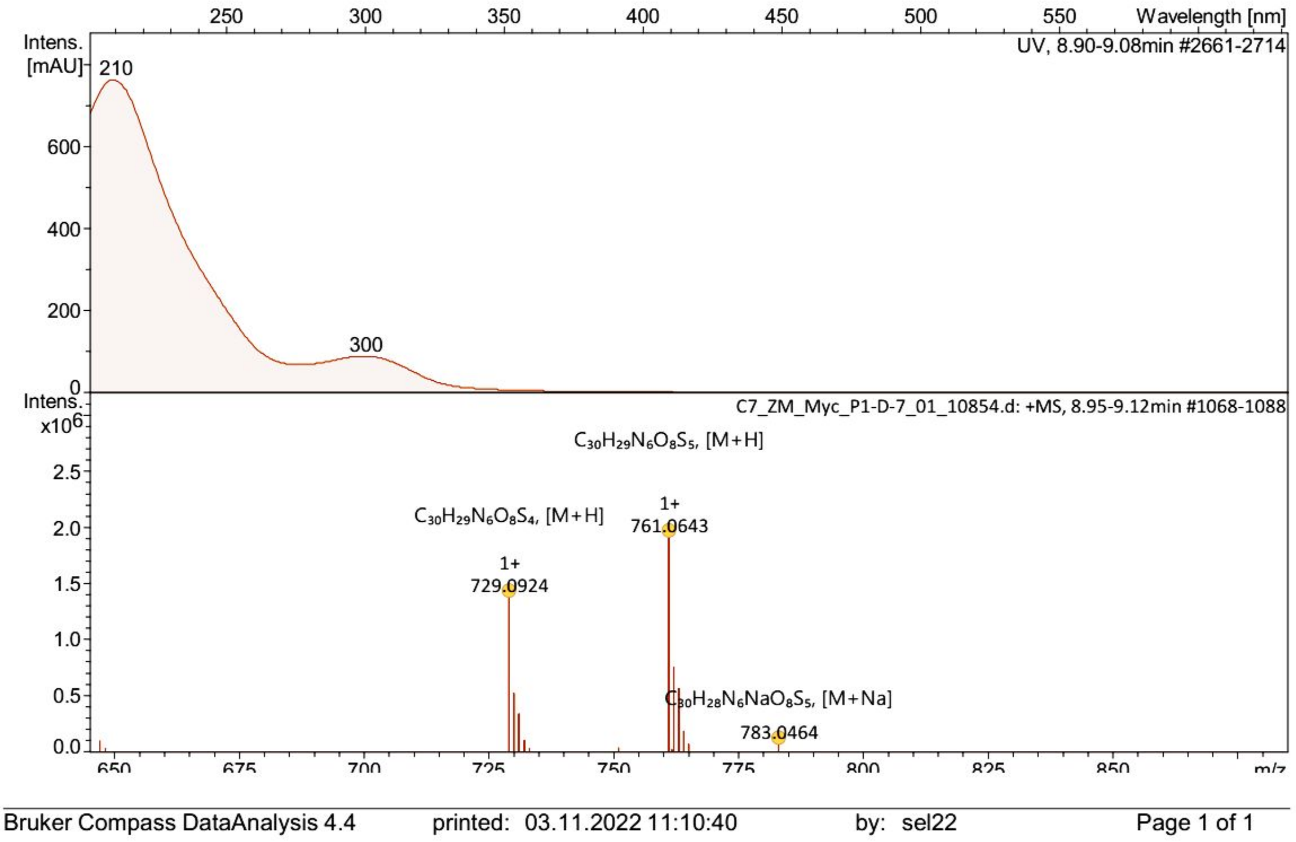

Figure S10. HRESIMS spectrum of **2**.

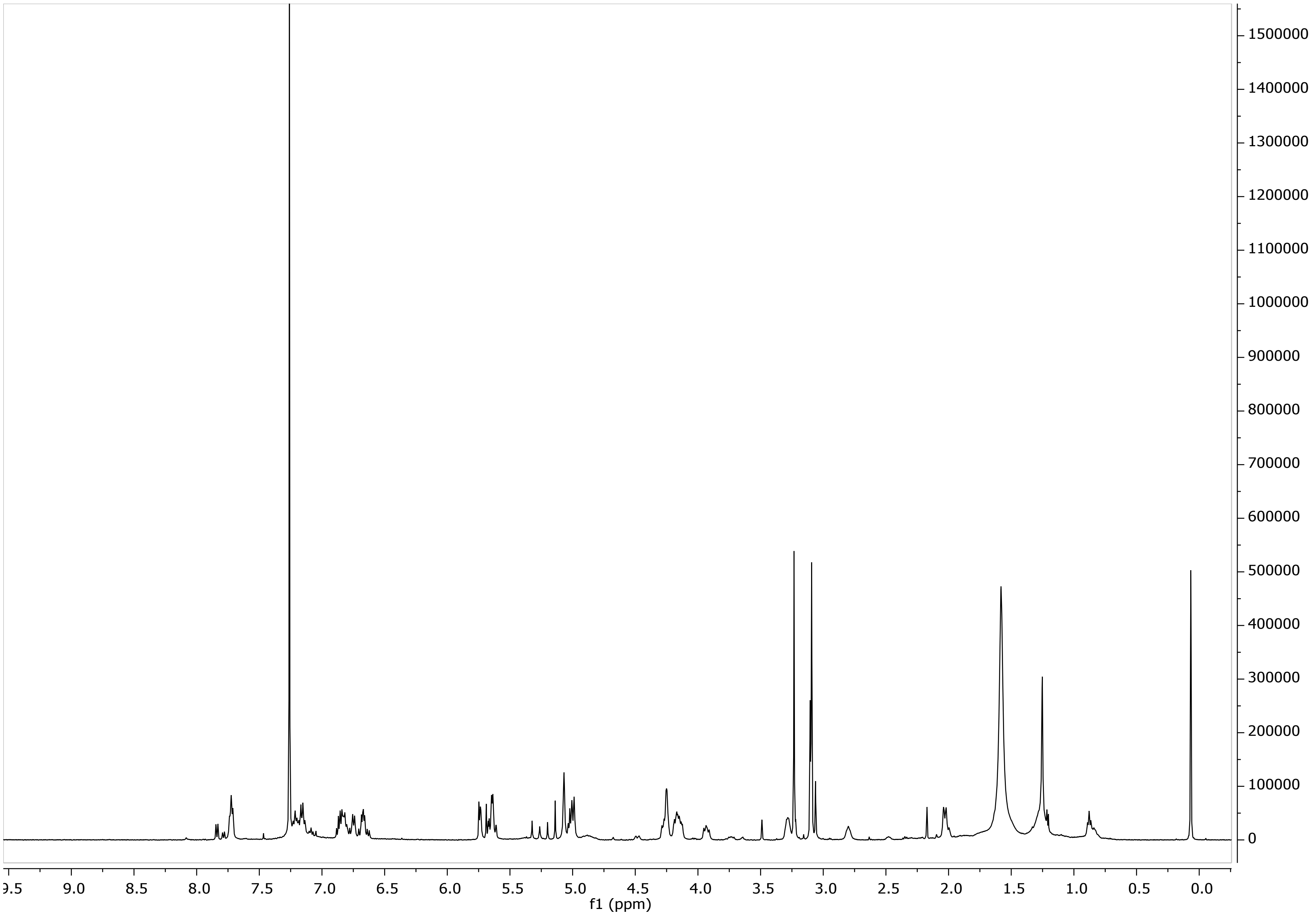

Figure S11. ^{1}H NMR spectrum of **2** in chloroform-*d* at 500 MHz.

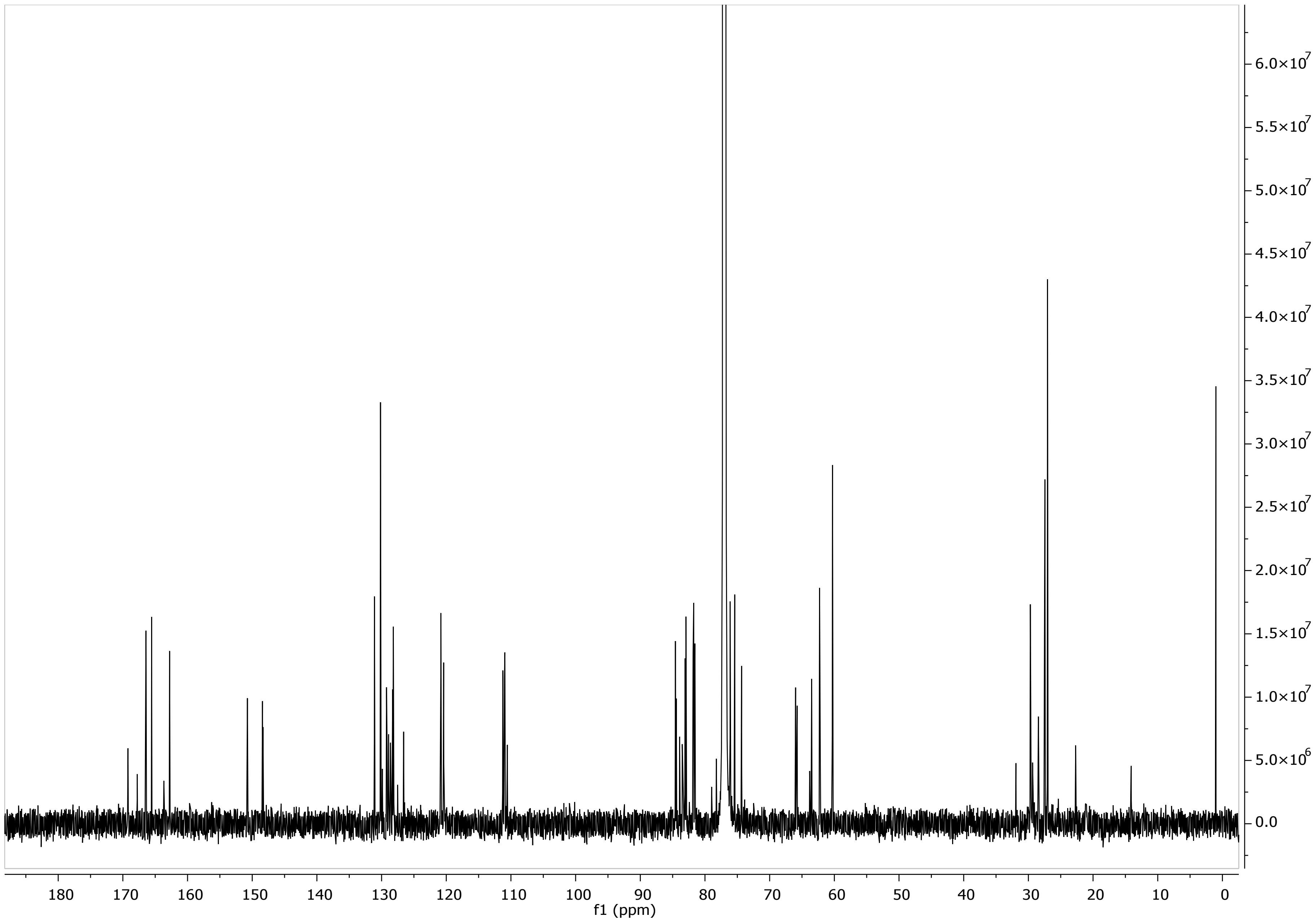

Figure S12. ^{13}C NMR spectrum of **2** in chloroform-*d* at 125 MHz.

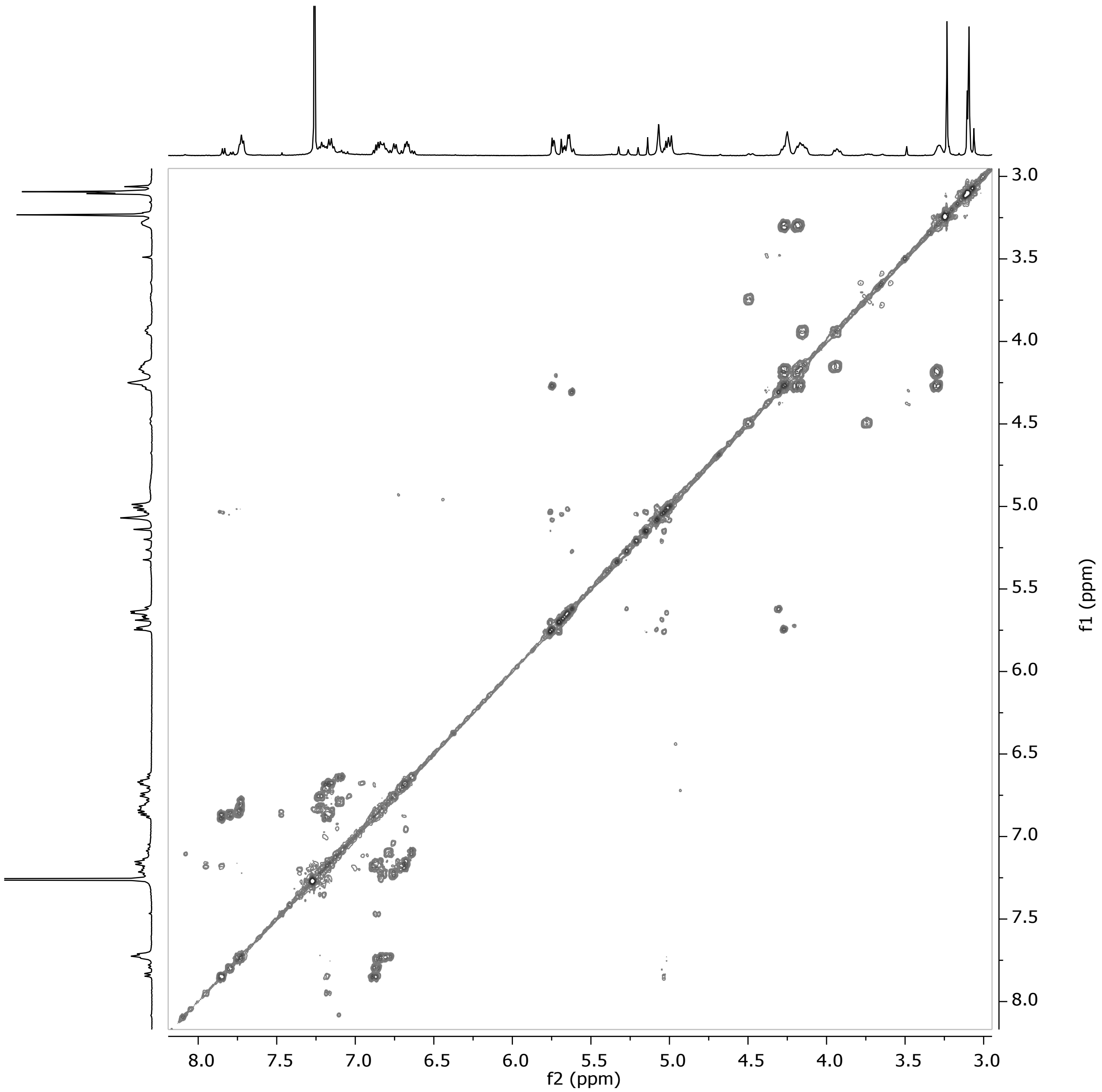

Figure S13. ^{1}H-^{1}H COSY spectrum of **2** in chloroform-*d* at 500 MHz.

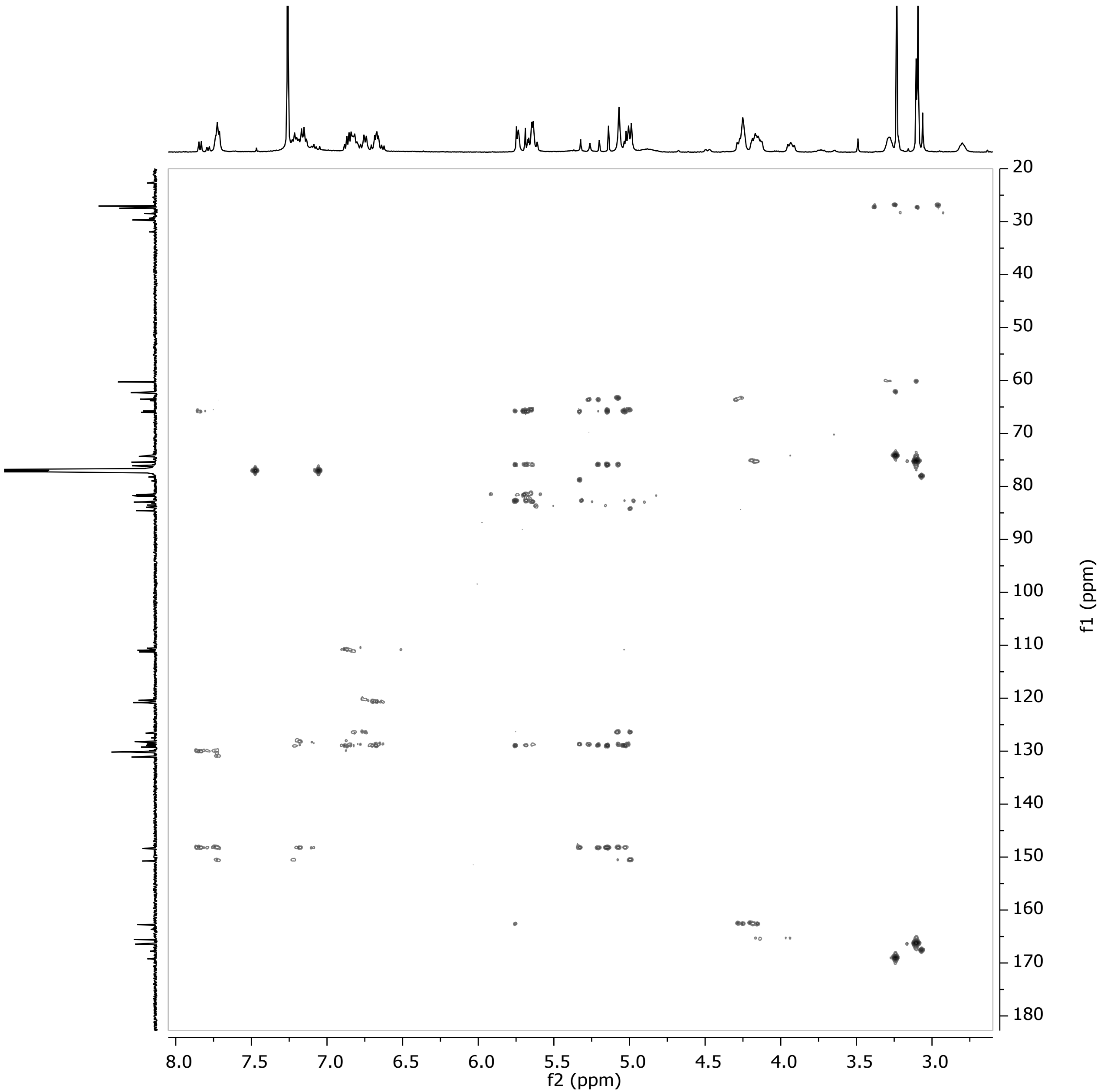

Figure S14. HMBC spectrum of **2** in chloroform-*d* at 500 MHz.

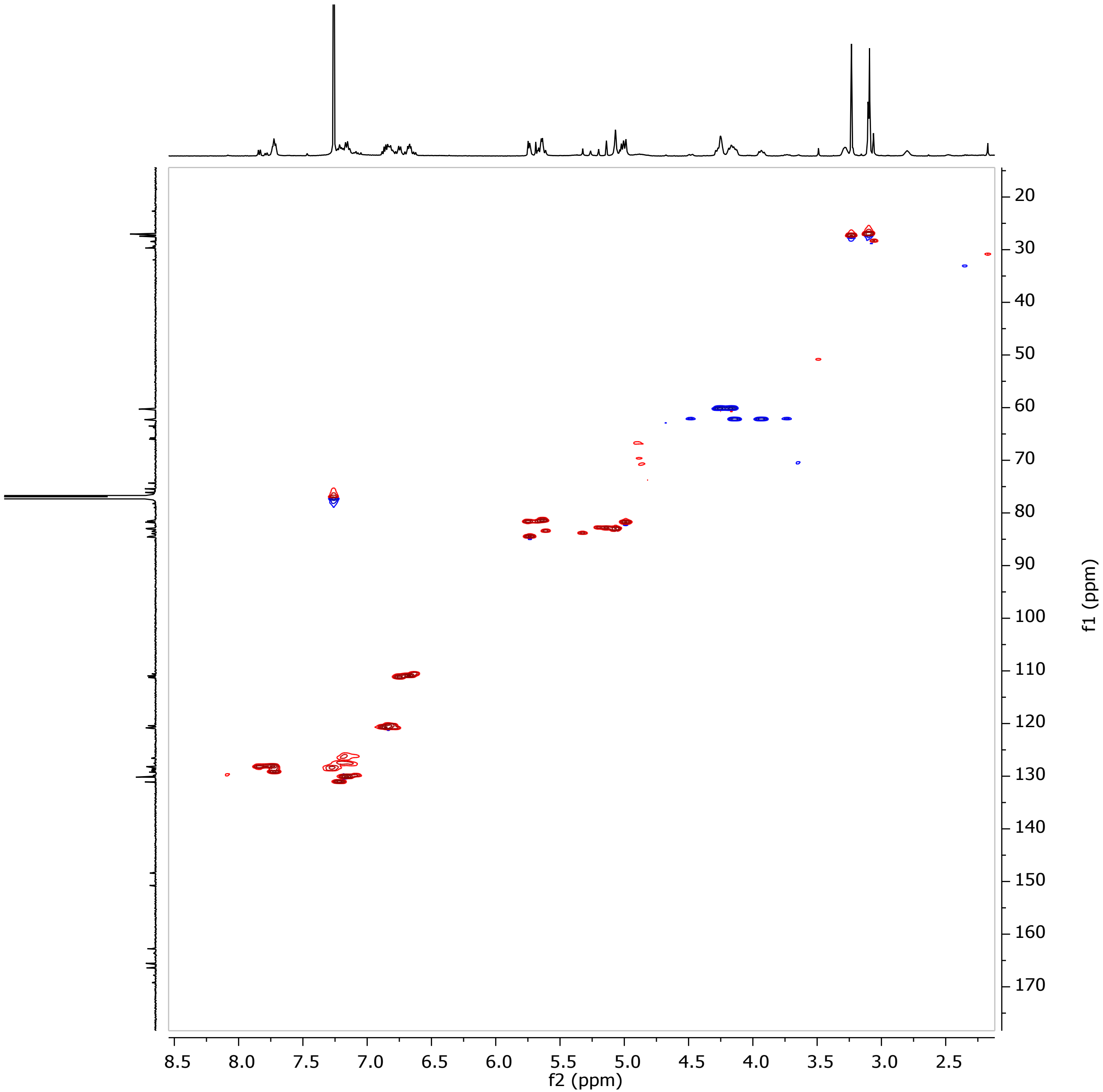

Figure S15. HSQC spectrum of **2** in chloroform-*d* at 500 MHz.

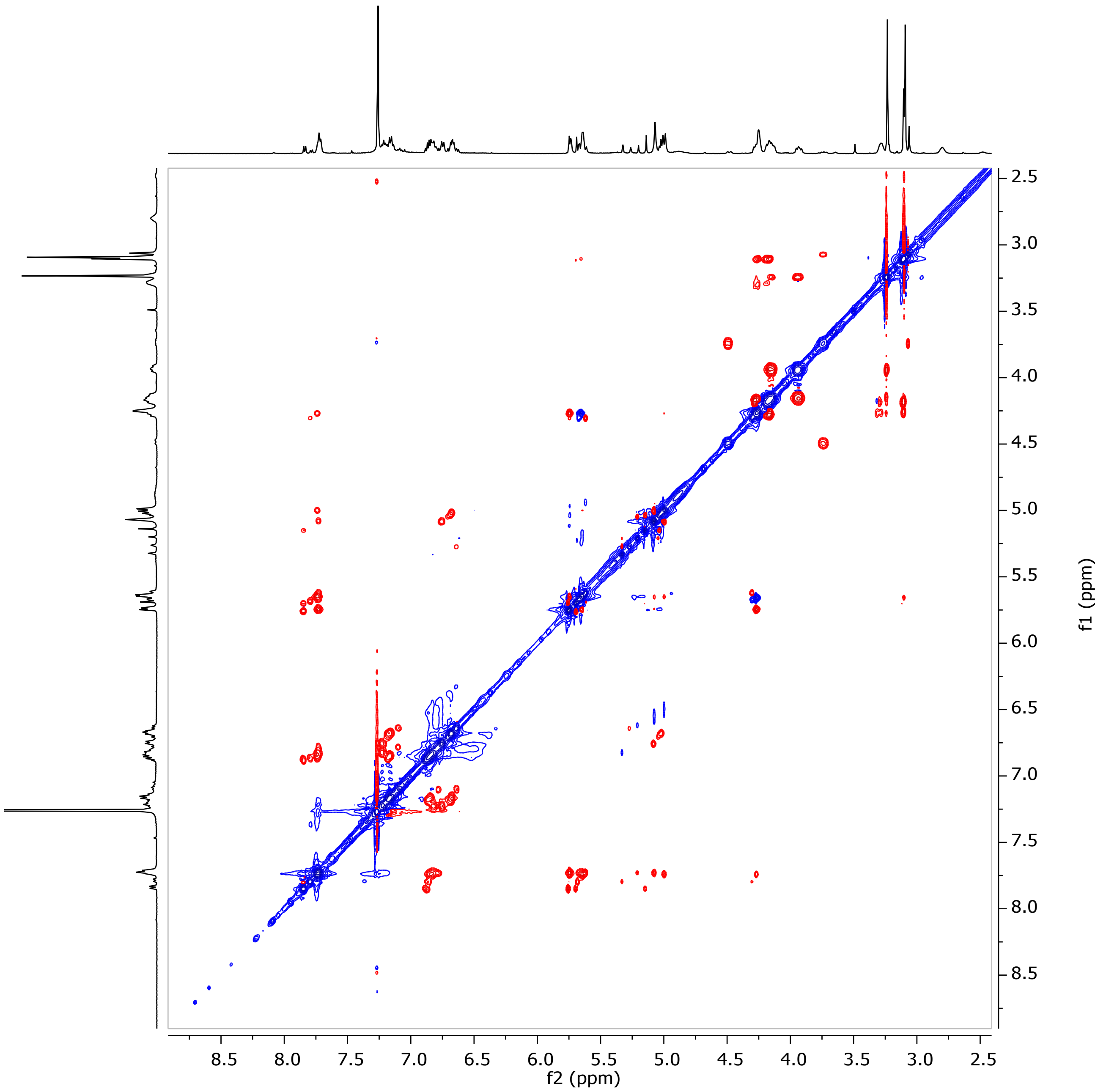

Figure S16. ROESY spectrum of **2** in chloroform-*d* at 500 MHz.
